# Audiovisual teleconsultation for patients with epilepsy in primary care in rural Germany: a pilot study on feasibility and acceptance

**DOI:** 10.1186/s40814-022-01171-4

**Published:** 2022-09-21

**Authors:** Gregor Feldmeier, Christin Löffler, Attila Altiner, Anja Wollny, Felix von Podewils, Manuela Ritzke

**Affiliations:** 1 Institute of General Practice, Rostock University Medical Center, Doberaner Str. 142, 18057 Rostock, Germany; 2 Department of Neurology, Epilepsy Center, University Medicine Greifswald, Greifswald, Germany

**Keywords:** Teleconsultation, Remote consultation, Telemedicine, Distance counselling, e-therapy, e-health, General practice, Family practice, General practitioner, Epilepsy, Neurologist

## Abstract

**Background:**

In rural areas, epilepsy patients have limited access to specialist secondary care. Substantial travel and waiting times of several hours are common. Communication between general practitioners (GP) and specialist epileptologists regarding diagnosis and treatment is further complicated by the high workload on both sides and the different prioritisation of treatment goals. This study aims to investigate the feasibility of an interprofessional audiovisual patient-doctor teleconsultation, and its acceptance in clinical practice in patients with epilepsy in a rural region in Germany.

**Method:**

Ten patients participated in telemedicine consultations in their GP practice. The practice was located in a sparsely populated region of Mecklenburg-Western Pomerania, and was equipped with technical equipment specifically procured for the project. An explorative qualitative interview was conducted with all participants. We based this paper on the consolidated criteria for reporting qualitative research (COREQ).

**Results:**

Despite initial uncertainties on the patients’ side regarding the consultation setting, all participants found the teleconsultation helpful. Some patients were initially intimidated and felt slightly overwhelmed by the attention provided and the technology used (multiple HD cameras, large high-resolution screens). However, during the consultation, they felt supported by their GP and were satisfied that their needs were addressed in an appropriate and timely manner. The hardware used was not felt to be a nuisance or to interfere with the conversation between doctor and patient. Patients also appreciated the time saved and the organisational convenience compared to a visit to a university outpatient clinic. Most consultations led to therapeutic consequences. Some patients seemed to benefit particularly, for example those who needed a medication change.

**Conclusion:**

This pilot study provides first evidence that teleconsultations between patients, specialists, and GPs are possible in rural areas. Interprofessional collaboration between GPs and epileptologists can improve the care of patients with epilepsy. Further research should investigate the effectiveness and efficiency of interprofessional telemedicine consultations for epilepsy and other conditions.

## Key messages regarding feasibility

- What uncertainties existed regarding the feasibility?
  It was unclear to what extent patients would accept the new form of consultation with simultaneous presence of patient, GP, and specialist.
- What are the key feasibility findings?
  The pilot study showed that patients quickly adapted to the new situation of the teleconsultation and were satisfied with the consultation outcome. The consultation model is suitable both for an initial contact with the epileptologist when epilepsy is suspected, and for specialist follow-up care.
- What are the implications of the feasibility findings for the design of the main study?
  For the design of the main study a uniform appointment management, and a documentation and billing system should be established.

## Background

The hidden nature of epilepsy poses a challenge to optimal health care for patients globally [1]. Disparities in treatment are described within countries, with a greater treatment gap in rural areas compared to urban areas. This is attributed to a combination of inadequate access to services, stigma and negative beliefs toward epilepsy, and low health literacy of the population [2].

Treatment strategies seem to be different in rural areas. For example, in rural areas of Germany, different medications are prescribed for specific underlying neurological diseases compared to urban areas in the same region. This is possibly a consequence of a lack of experts in rural areas [3]. Further, travel time for patients, who tend to be immobile, and costs for visiting a specialist play an increasingly important role. With regard to climate change, it is important to reduce unnecessary journeys.

General practitioners (GPs) are the first point of contact for medical care of patients in Germany. They cover basic medical care on the basis of a long-term doctor-patient relationship, as well as primary medical care. GPs take over the care and also manage further outpatient and inpatient treatment steps: they are also called as gate openers or gate keepers of the health care system. The pivotal role of GPs means that implementing telemedicine in their work can particularly contribute to improving health provision.

In their systematic review, Wootton et al. use the example of tele-dermatology to showcase potentially avoidable visits to the doctor. The average percentage of avoided trips reported in the 12 store-and-forward studies (this means the collection, closed forwarding to experts, and subsequent evaluation of patient data) was 43%. In the seven real-time studies and in a single study using a hybrid technique, journeys could be avoided for 70% of patients [4].

Average patient travel times for a visit to a neurologist in rural regions of Germany are 42 min by car. An added factor in our patient population is the common incompatibility of epilepsy medication with driving. Thus, transport accessibility plays an important role. Only about 62% of insured patients reported using a car to visit their doctors and public transport increases the travel time for the remaining patients [5]. Another study compared access to the GP with that of three exemplary specialists by public transport in a rural region of Germany. For example, a return trip to the GP averages 99 min compared to 160 min to the urologist. 6.5% of the inhabitants of the investigated district had no access to internal medicine specialists by public transport [6]. Based on the findings the same likely applies to neurologists.

Furthermore, considering the demographic change and the increased percentage of elderly patients, disease patterns and specialist care needs are going to change. For example, the incidence of age-related degenerative brain diseases, such as Parkinson’s and dementia [7], has already increased and will continue to do so in the future. Therefore, we need new concepts to counter this development.

In our study, the connection of GPs and neurologists was part of a wider project of establishing a telemedical connection of the Greifswald University Medical Centre neurology department with other medical providers. Smaller hospitals in the region were also integrated. This pilot study emerged from an initial sandpit meeting between neurologists experienced in telemedicine, and GPs, who are still inexperienced in this field. As this revealed motivation for further cooperation, we developed an intervention based on the theoretical framework “Six steps in quality intervention development” (6SQuID) by Wight, being a practical guide aimed at developing social interventions to improve public health [8]. The focus of this paper is the presentation of the practical implementation and the results regarding the fifth and sixth steps of this guide: testing and fine-tuning an intervention on a small scale, and gathering sufficient evidence of effectiveness to justify a more complex intervention.

In summary, this pilot study aims to test the feasibility and acceptance of an interprofessional audiovisual doctor-patient teleconsultation for epilepsy patients in rural regions of Germany. Application of the intervention is focused on the primary care sector of a rural area, and in particular the general practice. Possible indications for the improvement of the cooperation between GPs and specialist neurologists in regions with deficient medical provision will be addressed. Investigating the acceptance of innovative patient-centred care, for example through telemedicine consultations, for selected neurological diseases, such as epilepsy, is a further research interest.

To further develop this form of health care, it is vital to consider opportunities and limits explored from the perspectives of all involved groups (patients, neurologists, GPs). In agreement with the study design, the main focus of this paper is the patients’ perspective, but aspects from the physician's perspective have already been collected. These analyses are also presented.

## Methods

GF, who works as a research scientist and practicing GP is the first author of this article and developed the intervention in cooperation with a hospital-based neurologist (FvP), an expert in epileptology, and head of the Epilepsy Centre at the Greifswald University Medical Centre. The research associate and co-author MR, a diploma pedagogue with expertise in qualitative social research including the field of general medicine, carried out the qualitative process evaluation according to the framework of this pilot study. GF and MR work at the Institute of General Practice at the University Medical Centre Rostock. MR was not involved in the preparation, organisation, and testing of the intervention in one GP practice.

Ethical approval for this study was obtained from the Ethics committee at Rostock University Medical Center (Ref: A2018-0143). The only data collected within this study took place in the context of the process evaluation and the interviews conducted by MR for this purpose. No clinical data beyond routine care were collected or passed on to third parties by the study doctors. In addition, no video recordings of the teleconsultations were made.

### Design

We first implemented and tested the technical equipment required for a telemedical consultation with a neurologist in a family practice. We then developed a concept for audiovisual GP-specialist-patient teleconsultations and integrated it in the GP’s conventional daily care routine. The chosen sample size was based on the sample size policy for qualitative studies using interviews (*n* ≥ 5) [9]. Finally, 10 patients were included, which was also based on the self-assessment of the possible additional workload that could be managed by the GP. This number of participants was considered sufficient, not least for reasons of research economics.

To investigate feasibility and acceptability as part of the process evaluation, we chose an exploratory qualitative study design, as this allowed us to show both feasibility and possible barriers of the telemedicine service. We interviewed the GP, the neurologist, and 5 of the 10 participating patients to understand their experiences. We used the consolidated criteria for reporting qualitative research (COREQ) for reporting this study [10].

### Setting

We conducted the pilot study in Mecklenburg-Western Pomerania, a predominantly rural region in North-Eastern Germany. In a nationwide comparison it is characterised by a high poverty risk rate [11] and underserved regions for medical services or regions at risk of medical undersupply [12]. The pilot study was performed in a primary care group practice supplied with technology appropriate for a teleconsultation. The selected patients took part in a teleconsultation with their GP and a specialist from an epilepsy outpatient clinic at the Epilepsy Centre of the Greifswald University Medical Centre. As a tertiary epilepsy centre, it is responsible for a population of more than 500,000 people.

### The pilot study

Participating patients had a confirmed or suspected diagnosis of epilepsy. Patients who would have normally received a referral to a neurologist during their GP consultation met the inclusion criteria for the pilot study. Ten patients were recruited.

The technical solution required to enable teleconsultation in the GP's consulting room had to guarantee both a stable data connection for the audiovisual signal, and an end-to-end encryption. We relied on HD cameras and large high-resolution screens, which offered the best possible image transmission. Technical solutions from the company MEYTEC were used. Next, the premises of the GP practice had to be designed so that the patient and GP could communicate optimally with each other, the conversation was optimally transmitted to the neurologist via video, and the patient and GP could see the neurologist on the screen.

The focus of the consultation was to build trust between patients and doctors to emphasise the “medically accompanying” character of the counselling, in the GP’s premises and together with the GP. The technical base station for telemedical care by the neurology department already existed, but had to be newly installed in the GP practice. The GP also coordinated the appointments with the patients and the neurologist. Patient history, imaging, laboratory parameters, and other technical preliminary examinations, such as previous EEGs, were transmitted to the neurologist in advance.

The teleconsultation was always initiated by the GP, followed by a short introduction of all participants. In their own words, patients provided information about the epilepsy syndrome, and semiology and frequency of the seizures, as well as previous and current drug therapies. The GP added information, for example individual observations, family history, and suspected diagnoses. The neurologist asked specific questions about the individual disorders and the effects of the previous therapeutic measures. In close consultation with the patients, possible further diagnostic steps or changes in medication were discussed. After the joint determination of further steps between patient, neurologist and GP, the teleconsultation was concluded. Finally, patients had the opportunity to ask the GP general questions or questions of understanding.

### Recruitment of participants

In total, there were 54 potentially eligible patients with epilepsy in the practice of the GP. The GP initially selected in the sense of a purposeful sampling ten of his patients with epilepsy, or suspected epilepsy which needed clarifying, as potential study participants. The practice software was used to create a list. He approached them individually based on the listet order. All of them gave written informed consent to participate in the telemedicine consultation and took part in the intervention. As the targeted number (*n* = 10) was thus reached, no further selection round was necessary. The patients’ very high motivation to participate in the study could be related to the high level of trust in the GP, but also indicates a primary openness to this novel form of care.

After the teleconsultation, patients were asked if they would also like to participate in a follow-up interview, to which five patients agreed. Shortly after the intervention, these patients were contacted by telephone by the female research associate (MR) to make an appointment for a telephone interview.

### Characteristics of participants

The age of participating patients (*n* = 10) ranged from 22 to 79 years. All of them lived in small towns in the rural county Vorpommern-Greifswald in Mecklenburg-Western Pomerania. Six patients had previously been diagnosed with epilepsy, and four patients were assessed with a suspected diagnosis during a previous consultation. A questionnaire was sent asking about the interest in participating. The participants spoke German and were considered to be mentally and physically healthy. One patient with a mental disability was assisted by his mother who is also his caregiver. One GP in private practice conducted and accompanied the doctor-patient consultations from the perspective of general practice. One hospital-based neurologist has contributed his expertise as an epileptologist.

### Qualitative data collection

The qualitative process evaluation of the pilot study took place between January and May 2018. Approximately 2 weeks after the teleconsultation, single narrative telephone interviews with a length of 5 to 30 min were conducted on one occasion by MR with the participating GP and the neurologist. There was no previous relationship between the interviewer and the neurologist. A sample of five patients was selected based on the ability to participate in an interview. One disabled person was assisted by their caregiver. Otherwise, the patients were interviewed without the presence of third parties and no one dropped out during the course of the study. There was no prior relationship between the interviewer and the interviewed patients. A total of three male and two female patients aged 22 to 64 years with heterogeneous social background were interviewed. Narrative interviews are well-suited to openly elevate individual experiences and to help researchers understand the personal attitudes and needs of study participants [13]. Initially, the following narrative stimulus was developed to elicit an open narrative in the patients:

*“I am interested in your experience with telemedicine care from your GP. Maybe you can just tell me about your last visit to your GP and what was different about it.”*

Following the main narrative, both internal and external questions were asked. Internal questions referred to the main narrative presented and served to deepen the narrated topics. With external questions, new topics were introduced by the interviewer to highlight specific research questions from the interviewee’s perspective. In our case, external questions related to expectations and concerns about the consultation.

We developed and applied the following stimulus for the interviews with physicians:

*“We are interested in your experiences with telemedicine care for your patients and what was different to usual.”*

If not mentioned by the physicians themselves, further external questions covered previous experiences with telemedicine, advantages from the doctor’s perspective, patient groups benefitting most from telemedicine, and options for continuation of treatment. Finally, demographic data on gender, age, and occupation were collected.

The interviews were audio-recorded, transcribed verbatim, and anonymised. A total of 141 min of audio material was recorded. The interviews with the doctors lasted between 23 and 70 min, and the interviews with the patients between 9 and 14 min each. Only the physicians took up the offer to view the transcripts.

### Analysis

Immediately after the interview, the interviewer wrote field notes in form of a memo, containing both information on the conduct of the interview and on its content [14]. These memos served as an initial analytical step in structuring thoughts, emotions, and associations of the interviewer regarding the experience of conducting the interview and impressions from the interviewees and their narratives. Next, a multidisciplinary analysis team, consisting of social and health scientists and physicians, analysed and coded the data on the basis of Mayring’s content analysis [15]. The interview material was initially processed by two coders (MR, GF). MR, GF, AW, CL, and AA contributed to the analysis and interpretation of the data set. Structuring the content of the interviews allowed to bundle and differentiate acceptance-promoting attitudes and experiences in relation to telemedical care at the GP from the perspective of all participating groups.

Based on this, category systems were created for each participant group using Microsoft Office. These category systems were developed inductively, and were refined several times, for example after discussions of the working group. When no more additional categories could be found, all remaining interviews were included in a category system. This system was thus completed deductively. Our final developed main categories, associated categories, subcategories, and codes are based on relevant text passages. They allow comparison of diverse experiences and evaluations. The participating physicians provided feedback on the results. Table 1 presents the category system developed based on patients’ experience and is divided into four main categories with associated categories and subcategories.

**Table 1:** Category system developed on the basis of the patient interviews

Main category	Category	Subcategory	Codes^**a**^
Preparation	Organisational preparation	Coordination of the appointment	1
		GP sends findings to neurologists	2
	Content-specific preparation	Patient education by GP	4
Expectations and concerns	Hopes and motivation to participate	Added value by drug expertise	4
		GP clarifies uncertainties	3
		More trust in GP than current neurologists	2
		Alleviation of drug side effects	1
	Concerns	Data security of video recording	1
		Adverse therapy changes	1
Perception	Initial experience	Insecurities	4
	Video and audio technology	Technical implementation	7
		Advantage over phone call	2
		Virtual communication	5
	Doctor-patient conversation	Trust-building	3
		Clear explanation	4
		Revision of prior medical history	3
		Discussing current medication	6
		Reducing fears and insecurity	3
Acceptance	Advantage in comparison to the specialist outpatient clinic	Time and travel savings for patients	10
		Suspected time savings for doctors	2
		Cost savings	1
		Organisational relief (caregivers/ working people)	3
	Assessment of the results	Increased patient safety	5
		Clarification of further therapy	8
		Improved access to specialist	2
		Improved QoL and state of health	3
	General assessment	Positive evaluation	7

^a^The codes refer to the number of times a subcategory was coded

The content analysis of the patient interviews contrasts with the more differentiated category system developed on the basis of the physicians’ narratives. For reasons of clarity, only the five main categories and associated key messages are presented in Table 2.

**Table 2:** Category system developed on the basis of the physician interviews

Main category	Associated key messages^**a**^
Previous experiences with audiovisual teleconsultation and outpatient needs	No experience of the GP
	Clinical experience of the neurologist
	Inadequate and uncertain current care situation of the target group
Expectations regarding outpatient telemedicine	Hopes of the GP for more patient and family safety and improvement of own medical care practice
	Neurologist’s desire for adequate treatment
	GP concerns about mental and social overload of insecure patients and patients in need of care
	Neurologist’s concern of a higher time expenditure
Implementation in the practice including preparation, results, and follow-up	Sensitive selection of suitable patients
	Most preparation effort on the part of the GP
	Moderation by GP, inclusion of all parties in case discussion, shared information exchange
	Initial diagnosis
	planning of further therapy and diagnostic steps
	anxiety and stress reducing measures
	Documentation and follow-up by GP and neurologist
Balancing and evaluation of results	Facilitated access to specialist care and improved rural supply
	Suitable for target group, possible follow-up with other patient groups with clinical findings
	Positive impact on doctor-patient relationship
	More transparent therapy planning for GP and patient
	Improved education and level of information for all parties
	Reassurance of the GP and family carers
	High acceptance by patients and relatives and clarity about positive conditions for acceptance
	Positive cost-benefit ratio in terms of time spent and effectiveness from neurologist’s perspective
Future challenges and wishes	Optimisation of scheduling
	Possible increase of the number of patients and diagnoses
	Implementation in acute care
	Simplified documentation and billability of the specialist consultation

^a^In contrast to Table 1, the focus here is on the main categories and associated key messages derived from the categories and subcategories

## Results

In the following, we summarise and contrast the results of the content analyses for both patients and physicians.

### Expectations and impressions of patients

None of the patients had any experience with telemedical consultation. The GP thus informed patients in advance about the procedure. Expectations and feelings about the upcoming new consultation type ranged from restlessness, tension, and indifference, up to positive expectations of the “video telephony”, which was perceived as modern. Overall, the hopes, for example, for the expected expert opinion about medication or the suspected epilepsy diagnosis, outweighed concerns. The following quotes show that the initial nervousness experienced by some patients subsided during the consultation:

*“... and I was, at that moment [preliminary talk with the GP] really restless, I have to say. Because I was excited about how this will proceed. But this unrest has suddenly disappeared [during the consultation].” (35 years old male patient)*

*“... from the very beginning somehow it was strange, that you are sitting in front of the GP but the specialist is also present. This was really weird, but you got used to it pretty fast. That fit in very fast. […] Well, you didn't really notice that you were practically telephoning over the Internet.” (32 years old male patient)*

This familiarisation effect to virtual communication was probably facilitated by the robust technical setup, and good sound and image quality. The technical solution seemed to recede into the background. The patients reported that a trustful relationship to the mainly unfamiliar specialist was immediately established.

However, one woman—the oldest interview participant—would have preferred direct contact.

*“... well it is different, isn’t it? Face to face contact is always better, but I found it quite okay. The specialist was very kind and you were able to see him really well. All in all, it was good, it is possible to do it this way.” (64 years old female care giver)*

### Final evaluation of patients

The patients emphasised the high expenditure of time a specialist outpatient clinic visit would normally require. This includes the long journey of up to 1.5 h each way, but also the waiting time on-site. One patient highlighted saving fuel costs, and another was happy not being forced to take a day leave from work. The disabled patient’s mother was particularly relieved about the significantly reduced organisational effort. Especially multimorbid patients and their caring relatives benefited from telecommunication, even more so if they lived in rural areas.

*“…the driving, then I would have had to accompany him [to the specialist], then I would have had to deregister him from the workshop for the disabled and all that. And then I would have had to wait. So, I found it quite good.” (64 years old female care giver)*

Patients were given ample space to tell their stories based on the neurologist’s inquiries, for example about the history of the disease. Patients perceived experiences with current medication and their possible side effects as a major topic. The neurologist even critically questioned long-standing therapies. This led to medication changes. One patient subsequently reported a significantly reduced seizure rate and intensity, and therefore an increased quality of life. For some patients, the need for hospitalisation for already suspected more in-depth diagnoses, was confirmed, and appointments were made.

One patient expressed his relief at having found a specialist on the one hand, with the GP also wanting to take over further coordination, e.g. of transport and medication, on the other. A very unsettled patient felt that his concerns were taken very seriously in the consultation and told us in the later interview that the rapid clarification of the suspected diagnosis via video conferencing led to prompt reassurance about future epileptic seizures:

*“... the video-conference really helped me. I became calmer, became more balanced. With the next epileptic seizure, I was more relaxed shortly after. (22 years old female patient)*

*“… it was my first experience with internet telephony. But it is a good thing! It has to be said quite frankly, it is super!“ (32 years old male patient)*

### Preparations and expectations of physicians

The epileptologist was already experienced in telemedicine. He emphasised that the consultations conducted with telemedical setting of this pilot study were very similar to the epilepsy consultation offered in his clinic.

The epileptologist’s main initial concerns concerned the ability to take a proper patient history as well as organisational and time efforts. However, doubts especially regarding the latter, were removed as he felt the telemedical consultation was well-prepared by the GP. In total, he invested on average 15–20 min of his time per consultation plus subsequent documentation. He said:

*“The colleague [GP] talks to the patients, prepares them for the fact that now someone from another clinic, whom the patients except for one, I think, do not know personally, will ask very specific questions, that he [epileptologist] is in the picture, that he already has medical findings.” (epileptologist)*

The GP first had to select suitable patients. He identified epilepsy patients who did not yet have a specialist at hand, although in his estimation this seemed necessary. Avoiding to disturb a well-functioning patient-specialist relationship by involving a different specialist in the teleconsultation was also important. However, especially for patients with inadequate medication or those with a poor relationship to their neurologist, he saw major advantages of this type of teleconsultation.

In his view, uncertainties in diagnoses and treatment could be clarified rapidly with major advantages for patients. This regarded, for instance, his wish for a second opinion on a patient’s unspecific symptoms that seemed to indicate epilepsy.

As to coordination of appointments, the GP experienced an increase in workload. Clearly, the coordination of three parties involved more effort than the coordination of an appointment only between two parties. The GP was particularly concerned about the presence of the disabled patient's mother, whom he had experienced as dominant in conversations especially since the care relationship with this family had not existed for long. In this context, he also expressed concerns about whether the neurologist, who was virtually connected, could be additionally challenged if another fourth person commented on the patient's case in a potentially space-occupying way. However, during the teleconsultation these concerns turned out to be unfounded. She adapted to this new form of consultation quickly and seemed to be relieved and reassured afterwards. From his point of view, the relationship of trust between him and her, but also with the patient, has improved as an important result.

The GPs’ positive perception coincides with the statements of the patients. Overall, the GP found the pre- and post-processing work manageable, although he had the additional effort of copying the preliminary findings and sent them to the neurologist for preparation. Prior familiarity with patients was an advantage. Follow-up was not different from conventional consultations—rather the neurologist’s additional consultation report provided more comprehensive documentation for the patient record.

The patient encounter could not be saved as a record in the hospital information system, a problem for the specialist as the counter could not be billed. At the time of the pilot study, there were no billing modalities for a teleconsultation between a GP and an off-site specialist. In Germany, medical services are billed via a standardised evaluation scale, which provides expedient and economic care for people with statutory health insurance. Future implementations need to solve this problem. Acute cases and very short-term appointments are a further major challenge for this type of teleconsultation due to organisational issues. The neurologist saw limited use for patients requiring a detailed neurological examination, for example those with Parkinson’s disease, other movement disorders, or multiple sclerosis. These cases would exceed the time frame and the expertise of the GP, and still require personal face-to-face appointments in a specialist clinic.

### Final evaluation of physicians

The neurologist emphasised that telemedicine is very well suited for epilepsy patients, a diagnosis that heavily relies on the medical history. In this view, teleconsultation would also be suitable for patients with other diseases, who have already been presented to his or another specialist department, have an up-to-date examination result or a clear treatment plan; patients, in other words, for whom the focus is more on follow-up care.

*“Well, I’m thinking for example of stroke patients, who might at some point have to deal with the question of adjusting blood thinning, adjusting the risk factors, which means drugs that influence the risk factors, or patients, who may have been seen by a neurologist with a Parkinson's syndrome and where the point is that if you have an intolerance to the medication, you can perhaps decide to reduce the medication or switch to another one.” (epileptologist)*

The neurologist described the specialist care in the pilot GP’s region as insufficient with appointments at his epilepsy clinic being very difficult to obtain. This is consistent with the experiences of the interviewed patients. The specialist was convinced that teleconsultations facilitate making timely appointments with a simple phone call from the GP. He suspected that some of the study participants would not have visited a specialist outpatient clinic of their own accord. From his point of view, the familiar GP could be a door opener, with the offer of teleconsultations lowering the inhibition threshold of hesitant patients.

The GP saw great added value in teleconsultations, especially at the level of doctor-patient-communication but also the communication between doctors. Our proposed format would thus strengthen a relationship of trust with the patient and family members involved in the care. Above all, he felt like a partner of the neurologist; in each teleconsultation, all three participants communicated with each other at eye level. He saw himself first and foremost as a moderator who supports patients in describing their complaints and symptoms to the specialist and formulating their concerns. After the consultation, the GP summarised topics of discussion and plans for proceeding for the patient.

In some cases, previously unrecognised problems and knowledge gaps became apparent. These could be discussed and clarified. In this respect, the teleconsultation allowed the patient, GP, and specialist to be on the same level of knowledge. This is rarely the case in the German health system, where patients with chronic illness or multiple comorbidities consult a multitude of different specialists. The specialist’s targeted questions provided the GP with further information about the patients. This was particularly beneficial for the GP’s patient-centred treatment approach.

GP uncertainties regarding further treatment could also be clarified quickly. Necessary therapy steps or adjustments such as hospital admissions or medication changes were initiated. According to the neurologist, a brief specialist assessment via the telemedical consultation is a good way of making simple recommendations to the GP and clarifying whether action needs to be taken quickly. To implement teleconsultations on a wider scale—for example for other diseases—the GP hopes for a system that coordinates appointments. In summary, despite the individual challenges both the GP and the neurologist experienced teleconsultation as enriching.

*“And I see the added value in the fact that, if you have such a form of access to a specialist, you can choose the time together with the patient and here you can have a much more thorough and more profound form of consultation, if the three of us carry out the whole thing, which is where I see the great benefit and added value in the situation.” (GP)*

From the neurologist’s point of view, the future expansion of this cooperative form of consultation is feasible and worthwhile for all sides involved. Adjustments to the current concept of telemedicine should also be considered.

*“That’s [teleconsultation] something we'll be able to absolutely master if it continues on this scale. It can also be expanded. It's not the mass of patients you see here now and it's not very frequent, regular appointments. Hm and if not, then you just have to think about other concepts, personnel concepts. But the way it works now, it works very well and the decisive thing is that the patients benefit from it. And the second point is that both sides, the general practitioner and us, profit from it. And I think that's also the way we go about it.” (epileptologist)*

## Discussion

### Summary

Overall, patients accepted the offer of a teleconsultation very well and experienced it as positive. The novel conditions required initial familiarisation. The trusting environment and the stable relationship with the GP supported this. With the help of the GP, patients were able to mentally prepare themselves for the teleconsultation. This further increased acceptance. Both the technical implementation of teleconsultation and the form of communication were perceived by the patients as so self-evident that no criticism was voiced in this regard. Quantitative consequences regarding the therapy regime or further diagnostic measures were not the focus of this feasibility study.

### Comparison with existing research

Previous research shows that patients who use teleconsultation often report some mistrust in data security [16]. In our pilot study, however, only one patient expressed uncertainty about the whereabouts of the video footage. In previous studies patients were unsure about the outcome of the consultation and possible changes in therapy. This was often related to patients not knowing the specialist beforehand. Our pilot study showed that patients particularly valued the time and cost savings of teleconsultation. Other studies also confirm this [17]. These advantages were also of particular benefit to accompanying relatives of patients in need of care, all the more so if they are employed.

The working group of Salisbury and colleagues investigated so-called private video-first consultation services for primary care in the UK and found high levels of satisfaction especially among young and healthy patients [18]. This satisfaction could be an impetus to include teleconsultation in the GP care portfolio also in Germany.

Further positive effects for GPs were found in Tönnies and colleagues’ survey on the implementation of video consultations in general practice. Performing a qualitative cross-sectional study, they conducted 15 semi-structured interviews with health policy experts representing different interest groups of the German health system: health insurance funds, government agencies, professional associations of clinicians, and patient representatives. Using psychiatric specialists as an example, the study showed that health policy stakeholders perceive video consultations as a way to improve primary care. Overall, the interviewees mentioned advantages for GPs in terms of relief through saving time, the possibility of referring patients to psychosocial treatment, and lower-threshold access on the patient side [19].

To facilitate the introduction of future teleconsultation models with specialists in GP practices, the working group Hoffmann and colleagues developed a checklist, using specialists in psychiatry as an example. Their checklist agrees with the findings from our study. Hoffmann et al. summarise the need for concrete clarification of responsibilities and tasks, involving patients in a trusting manner, structured consultation hours, and cooperation between GPs and specialists [20]. Especially the latter is important as teleconsultation may increase interprofessional exchange. This enables the joint coordination of diagnosis and therapy.

### Strengths and weaknesses

The present pilot study was conducted in a rural and structurally weak region with a low density of neurologists. There was a clear need for teleconsultation with a neurological expert. The chosen approach of bringing together GP, patient, and specialist in one consultation enables a direct interprofessional exchange, which is uncommon in the German health care system in this form. The chosen way of implementation highlights the patient’s point of view through a patient-centred approach. From the neurologist’s perspective, the teleconsultation was comparable in content to the outpatient epilepsy consultation in the hospital. For the patient in particular, there was the possibility to quickly clarify open questions about diagnosis and therapy and to coordinate further steps immediately with the GP and the specialists. Both the GP’s perspective and the specialist's perspective could be taken into account.

One weakness of this study is that only one GP and one neurologist carried out the pilot of this intervention: there was no previous experience with this form of cooperation on either side, and time and financial effort were initially not foreseeable. Thus, the pilot study, focusing on basic feasibility and practicability of teleconsultation in general practice, had to start at a low level to pave the way for a more complex intervention, and possibly broader resource funding. Moreover, both physicians had a high technical affinity and were intrinsically highly motivated to optimally conduct the pilot study. This motivation and the already existing technological literacy may not be transferable to this extent to a larger-scale study.

Another limitation is that the GP had the role of both the GP therapist and the study initiator and coordinator. The objective assessment of the intervention process was realised with a methodically controlled process evaluation by a third party.

Our results confirm that this form of counselling is time-intensive. In a larger-scale study, opportunities for minimising time expenditure should be considered, for example through external appointment coordination.

### Implications and need for further research

The chosen approach offered patients in Germany the opportunity to simultaneously consult their GP and a specialist about their epilepsy for the first time. This improved the doctor-patient relationship and contributed to patient empowerment, for example through possible support of the GP in describing patient-related health problems. Decisions could be made jointly by taking into account the perspective of all three parties. This created a completely new form of a partnership-based doctor-patient contact. The targeted preparation of the discussion by the GP helped to implement the exchange with the neurologist in a time-efficient manner.

The organisation of the consultation emerged as the biggest hurdle: both the coordination of the patients in the normal consultation hour and the time coordination with the neurologist required an organisational effort. However, the added value of a targeted and comprehensive interprofessional exchange between different disciplines could compensate for the organisational effort of the GP. The technological set-up is mature and was used without any problems throughout the pilot study. The patients quickly adapted to the changed conversation situation. However, as the necessary technical equipment in GP practices can vary and a variability in technological literacy must be assumed, costs for technical equipment and the need for training courses should be explored in further studies.

The optimisation of the organisational processes in the practices and clinics should also be the focus of future research work. Further required studies include a numerically larger feasibility study with accompanying evaluation, and a randomised controlled trial.

## Conclusion

This pilot study provided initial evidence that teleconsultations between patients, specialists, and GPs in rural areas are feasible and useful. The use of teleconsultations for selective disease patterns, such as epilepsy in this case, enabled efficient consultations close to the patient’s home. Other possible applications could be found, for example, in patients with Parkinson’s disease or Guillain-Barré syndrome, who are also limited by immobility. Especially in rural areas with a low density of specialists, this approach can reduce future problems in health care.

This pilot study only provided evidence for the general feasibility on a limited scale. An understanding of long-term efficiency and effectiveness from several GP practices is still lacking and can only be obtained through a larger-scale feasibility study and randomised controlled trial. Within the framework of such a study, a uniform appointment management, documentation, and billing system should also be established. This could minimise preparation time, possibly enabling time and cost savings. After all, these factors will be decisive in determining the extent to which specialist teleconsultation can be integrated into the future daily routine of GPs.

## Data Availability

The datasets used and/or analysed during the current study are available from the corresponding author on reasonable request.

## Abbreviations

GP: General practitioner
QoL: Quality of life
